# Three-dimensional cellular dynamics and mandibular morphogenesis

**DOI:** 10.3389/fcell.2026.1823297

**Published:** 2026-05-18

**Authors:** Andreas Dauter, Sofia K. Malhi, Lucas D. Lo Vercio, Elizabeth C. Barretto, Kim M. T. Nguyen, Cassidy Da Silva, Alejandro Gutierrez, Ralph S. Marcucio, Benedikt Hallgrímsson

**Affiliations:** 1 McCaig Institute for Bone and Joint Health, University of Calgary, Calgary, AB, Canada; 2 Alberta Children’s Hospital Research Institute, University of Calgary, Calgary, AB, Canada; 3 Department of Orthopaedic Surgery, University of California, San Francisco (UCSF), San Francisco, CA, United States; 4 Department of Cell Biology & Anatomy, Cumming School of Medicine, University of Calgary, Calgary, AB, Canada

**Keywords:** cell behaviours, craniofacial development, geometric morphometrics, light sheet fluorescence microscopy, mandible, morphogenesis

## Abstract

**Introduction:**

Shaping the face requires the coordination of molecular and cellular behaviours that direct tissue-wide dynamics of development. A limited, well-conserved suite of cell behaviours, including cell proliferation, facilitate cellular contributions to morphogenesis, which are in turn regulated by the tissues they comprise. However, the role of cell behaviours in early facial tissues remains poorly characterized, despite underlying both healthy and syndromic facial development.

**Methods:**

By integrating light-sheet fluorescence microscopy, shape quantification, and semi-automated image segmentation, we resolve individual cell behaviours and their relationships to morphogenesis across large tissue domains in the developing mandible. We focus on cell proliferation and mitotic orientation, but also analyze cellular morphology and cell-tissue relationships via positional orientation.

**Results:**

Here, we describe three-dimensional patterns of cell behaviours in the context of mandibular morphogenesis. We show that several cell behaviours are mediolaterally patterned in tandem at E10.5, a critical point in development. These patterns align with preceding growth, and occur several days before the onset of cell fate commitment.

**Discussion:**

These observations reflect the reciprocal interplay between molecular, cellular, and tissue-level processes, highlighting how subtle differences in cell behaviours are connected to complex growth patterns even in largely homogeneous tissues.

## 1 Introduction

The shaping of facial structures during development involves a cascade of integrated processes connecting molecular, cellular, and tissue-wide scales. Molecular processes inform cell behaviours, which in turn direct morphogenesis of complex structures. Some cell behaviours, such as oriented mitosis and migration, are inherently directional and often drive oriented patterns of outgrowth (Dokmegang et al., 2021; Green, 2022). Patterned morphogenesis is critical to the development of many tissues, and is facilitated by a limited but well-conserved suite of cell behaviours across all species (Boehm et al., 2010; Danescu et al., 2021; Green, 2022; Hopyan et al., 2011; Jülicher and Eaton, 2017; Li et al., 2013; Murillo-Rincón and Kaucka, 2020). Directional growth can also be facilitated by spatial patterns of cell behaviours, where processes like cell proliferation or cell death may show differences in localization regardless of orientation. These cell behaviours are coordinated by interactions between cells and their neighbouring environment, including morphogen signaling, adhesion patterning, cell polarity, and forces applied by the extracellular matrix and neighbouring cells (Davies, 2023; Niessen et al., 2011; Seilacher, 1991).

Oriented outgrowth is critical across all of mammalian development, and plays specific roles in developing craniofacial tissues. Directional processes are necessary to generate asymmetries, define proportions of a structure, establish differential regions of growth, and shape complex three-dimensional forms (Gao and Yang, 2013; Murillo-Rincón and Kaucka, 2020). These processes are particularly important in the early facial prominences, which rapidly remodel their shapes and proportions to establish the blueprint of the face. The cellular basis of outgrowth in facial morphogenesis, however, is poorly characterized. Studying the patterning of oriented cell behaviours may therefore provide valuable insights into both normal development and the etiology of structural birth defects.

Among directional cell behaviours in the face, oriented cell division (also referred to as mitotic orientation) is specifically highlighted in several mechanisms of morphogenesis (Lesnicar-Pucko et al., 2020; Poulson and Lechler, 2010). Measuring the contributions of mitotic orientation to directional growth is challenging due to the complex signaling and mechanical microenvironments in facial tissues (Bergstralh et al., 2017; Li et al., 2019; Nestor-Bergmann et al., 2014). The contributions of cell behaviours to mesenchymal tissue growth have best been modeled in the murine limb bud, in which oriented cell behaviours are necessary to drive directional growth of tissues which cannot be explained by spatial patterns of cell behaviours alone, such as localized proliferation (Boehm et al., 2010; Hopyan et al., 2011; Lesnicar-Pucko et al., 2020). Many fundamental processes of mesenchymal growth, including key signaling pathways, cell movements, and budding patterns, are conserved between face and limb bud development (Lee et al., 2004; Schneider et al., 1999; Tucker et al., 1999). Additionally, multiple cell behaviours may be spatially coordinated by shared processes, including mechanical or biochemical signals (Li et al., 2018; Lukashev and Werb, 1998). Quantifying the contributions of oriented cell divisions to facial morphogenesis is therefore imperative to understand the fundamental growth of mammalian faces.

Previously, we identified that small, diffuse differences in proliferation were associated with shape change in the facial prominences during embryogenesis (Lo Vercio et al., 2025). However, the observed differences in proliferation lacked directional patterning and did not align with the outgrowth of constituent facial tissues. Spatial differences in proliferation were important for large-scale tissue remodelling, but less important for driving directional outgrowth. In this study, we explore directional characteristics of cell divisions beyond spatial patterning. We hypothesized that spatiotemporal differences in the orientation of cell divisions are necessary for normal outgrowth of the facial prominences. To test this, we focused on outgrowth of the mandibular prominence (MdP) at embryonic day 10.5 (E10.5), which is composed of structurally uniform mesenchyme growing anteriorly. We imaged the division planes of mitotic cells in 3D and applied our previously developed tools to segment cells and tissues for further analysis. Next, we registered all samples to a common atlas to assess differences in the patterning of mitotic orientation throughout the MdP. Lastly, we employed geometric morphometrics to study the relationship between mitotic orientation and directional outgrowth. We found that even in structurally homogeneous tissues such as the MdP, cell behaviours organize in shared mediolateral domains, where their positional patterning aligns with preceding tissue growth. These patterns may contribute to large-scale changes such as jaw narrowing in facial structures.

## 2 Results

### 2.1 Light-sheet microscopy workflow for imaging mitotic spindle orientation

**FIGURE 1:**
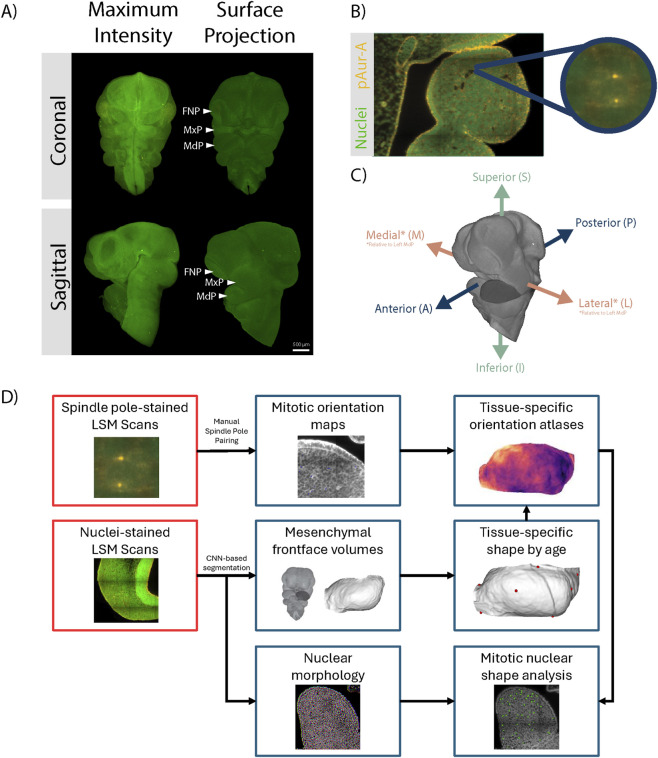
Light-sheet microscopy workflow for quantifying 3D cell behaviours. **(A)** Examples of 3D volumes of E10.5 embryos stained with Nuclear Green and generated using a Zeiss Lightsheet Z.1 microscope. The same image is shown as a maximum intensity projection (left) and a space-filling representation (right), which was used to quantify morphology. Single embryo, representative of n = 8 samples. **(B)** Single optical plane of an LSFM scan showing a representative pair of spindle poles (orange, phospho-Aurora **(A)** within a cell in the mandibular prominence (MdP). **(C)** Anatomical axes of an E10.5 mouse embryo and abbreviations used in this article. Lateral and Medial directions are established in relation to the left mandibular prominence (Dark Grey). **(D)** Overview of the workflow used to create shape and orientation atlases from LSFM scans. For some specimens (n = 4), nuclear shape was automatically segmented as flows using CellPose-SAM, while phospho-Aurora A-stained spindle poles are manually identified and paired. Mesenchymal volumes (n = 8) are generated for the mandibular prominence from surface projections of a nuclear stain, landmarked, and used to generate average shape atlases. These data were combined to create 3D maps of mitotic orientation and nuclear shape.

Light-sheet fluorescence microscopy (LSFM) allows the imaging of both individual cell dynamics and 3D tissue morphology in the same samples. Previously, we established an automated workflow for the segmentation, registration, and quantification of cell proliferation in LSFM images (Lo Vercio et al., 2022; 2025). Here, we modified this workflow to allow for the observation of directional cell behaviours in 3D space (Figure 1). We imaged four wildtype C57BL/6J mouse embryos between E10.0-E10.5, during which time the MdP initiates forward outgrowth(Higashiyama et al., 2025). From each scan, we generated volumetric images registered to a common space. To ensure these images were high enough resolution to accurately resolve sub-cellular structures such as the mitotic spindle, we imaged in a high-magnification 20x configuration. LSFM images are anisotropic, with the between-slice resolution 5 times lower than the in-slice resolution. As such, we did not pursue direct 3D segmentation, which could introduce directional errors based on the orientation of image capture. Instead, we captured samples in both the sagittal and coronal planes, identified in-plane divisions in the imaging plane of each, and transformed these into a common space. This allowed us to generate a pseudo-3D atlas of cell division for further analysis from 2D annotated images.

### 2.2 Cell divisions are aligned in the medial, but not lateral, mandibular prominence

**FIGURE 2:**
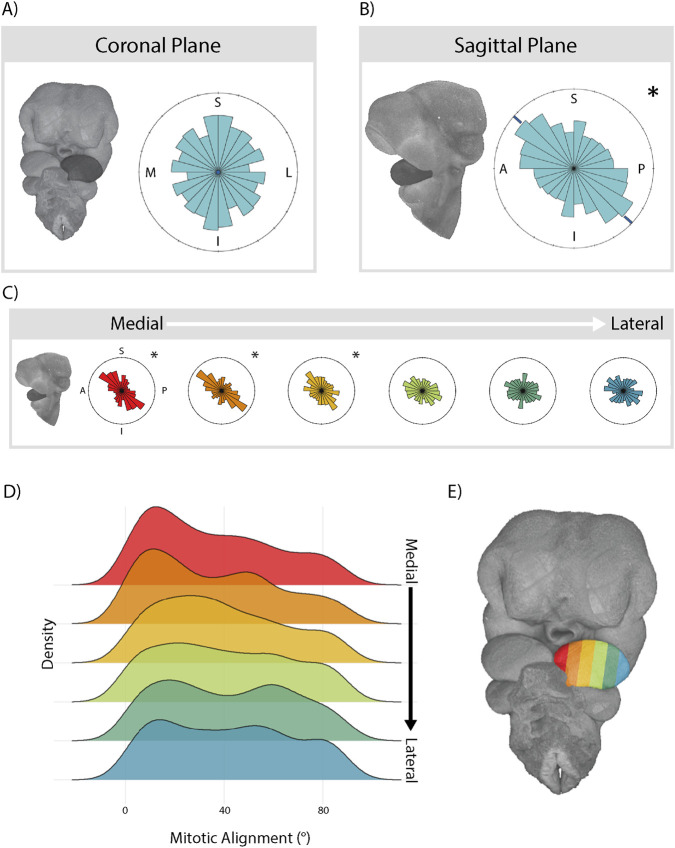
Cells in the medial MdP orient and align their divisions towards the anterior edge of the tissue. Rose plots showing total mitotic angle distributions in the MdP in the coronal **(A)** and sagittal **(B)** planes show that cell divisions preferentially orient anteriorly in the face. **(C)** Differences in mitotic angle across the mediolateral axis of the mandibular prominence, captured in the sagittal plane. Sections shown in 2E. Asterisks indicate significantnly non-uniform distributions of angles (circular Kuiper’s test) in all rose plots. **(D)** Ridge plots showing density of mitotic alignment scores for dividing cells along the mediolateral axis, where a lower score indicates a cell that aligns its mitotic orientation more closely to its neighbours. **(E)** Segmentation of the left MdP into six mediolateral sections used in C and D, as well as later figures. n = 1937 cells projected onto the sagittal plane and analyzed from N = 4 specimens.

First, we assessed mitotic orientation in both the sagittal and coronal planes to look for tissue-wide patterns of oriented cell division. Mitotic angles were coordinated in the sagittal plane, orienting towards the anterior surface of the MdP (Figure 2B). Conversely, cell divisions imaged in the coronal plane lacked any directional pattern (Figure 2A), suggesting that cell divisions are most strongly oriented along the dorsal-ventral axis in the MdP. To assess whether the oriented cell division was patterned locally or uniformly throughout the tissue, we analyzed patterns of cell division across the mediolateral axis by dividing the tissue into six segments. Cells in the three most medial segments of the MdP oriented their divisions towards the anterior surface of the MdP (Figure 2C). Conversely, mitotic angles of cells in the three lateral segments were randomly distributed. These observations suggest that positional cues within the MdP are associated with alignment of cell divisions, with well-aligned divisions in the medial tissue and poorly-aligned divisions in the lateral tissue. To quantify this, we calculated a ‘Mitotic alignment’ score for each cell, representing the difference between the cell’s mitotic angle and the average mitotic angle of all dividing cells within a 200 µm radius. A lower score represents a cell that orients its division more similarly to other neighbouring cells. Our results indicated that the medial domains in the MdP contained more well aligned (low scoring) cells than the lateral domains (Figure 2D).

### 2.3 Morphology of mitotic nuclei is more uniform in areas of higher mitotic alignment

**FIGURE 3:**
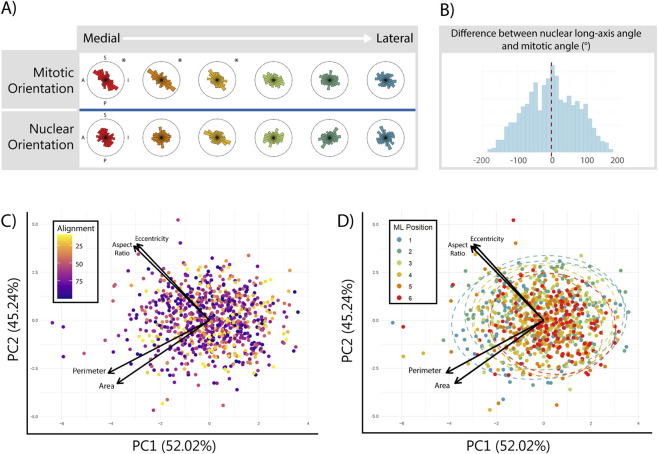
Nuclear geometry of mitotic cells in the MdP is more uniform in the medial tissue than the lateral tissue. **(A)** Rose plots showing the direction of spindle and nuclear long-axis orientations in the sagittal plane of mediolateral segments shown in Figure 2E. **(B)** Histogram showing difference between spindle and nuclear long-axis orientation for individual cells in the MdP. Mean difference (−2°) shown by a vertical dashed red line. PCA biplots showing the first two principal components of nuclear shape by mitotic alignment **(C)** and mediolateral position **(D)**. Arrows indicate each variable’s influence on the principal components. Ellipses in **(D)** represent 95% confidence intervals for variance of mitotic nuclear shape within each mediolateral segment (Watson’s U^2^ test). Nuclear shape quantification calculated from n = 4,520 cells from N = 4 speciments.

Cellular responses to extrinsic forces typically include changes to both surface and nuclear morphology (Goult et al., 2022; LeGoff and Lecuit, 2015; Miroshnikova and Wickström, 2022). As such, we studied geometric variation in dividing cells of the MdP to further understand the patterning of directional cell behaviours. For each dividing cell, we quantified five morphological features of the nucleus with CellProfiler: area, perimeter, aspect ratio, eccentricity, and long axis orientation. We aimed to understand whether positional differences in morphology were associated with positional differences in cell behaviours. First, we compared the long axis orientation of mitotic nuclei to their division angle. In all segments, nuclear orientation was more variable than division orientation and was only weakly correlated with mitotic orientation, suggesting that mitotic angle in facial mesenchyme is biased towards, but does not always follow, the cell’s long axis (Figures 3A,B). There was no correlation between any measure of nuclear morphology and mitotic alignment score, but eccentricity and aspect ratio were significantly correlated with mediolateral position (p < 0.001, Supplementary Table S1). To investigate higher-level variation in nuclear morphology, we conducted a Principal Components Analysis on all five measures of shape. Nuclei primarily varied in two directions: size (encompassing perimeter and area) and circularity (encompassing aspect ratio and eccentricity). There was no discernible relationship between mitotic alignment scores and any of the principal components of shape, indicating that the nuclei of cells that align their divisions with their neighbours are not morphologically distinct from those that divide in random orientations (Figure 3C). However, both nuclear size and nuclear shape became less variable and more uniform when moving medially along the MdP. The relationship between nuclear shape variation and position was significant along PCs 1 and 4, which explained 52.02% and 0.259% of the variation, respectively (p = 0.0002, p = 0.001).

### 2.4 Mitotic alignment follows directional growth in the developing jaw

**FIGURE 4:**
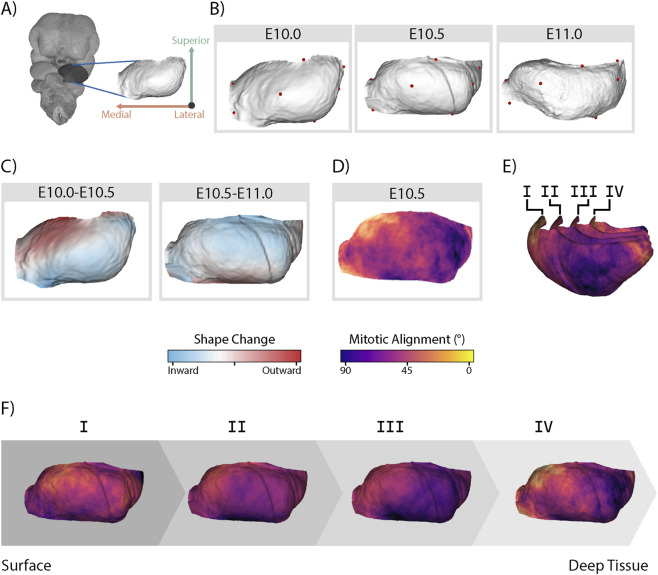
Shape change precedes patterned mitotic alignment in the MdP. **(A)** Segmentation of the MdP surface from each volume. Surfaces were averaged to create an average atlas of each age point. **(B)** Mean shapes, including landmarks, of the left MdP at E10.0 (n = 10), E10.5 (n = 10), and E11.0 (n = 8). **(C)** Shape change of the left MdP from E10.0-E10.5 and E10.5 to E11.0 scaled to unit size with Procrustes superimposition. Red indicates regions that are outwardly displaced in the later time point compared to the earlier time point, while blue indicates inwardly displaced areas. **(D)** Average mitotic alignment of cells within 200 µm of each point across the E10.5 left mandibular surface. (n = 4,520 cells from N = 4 specimens) **(E)** Alignment scores of nearby cells calculated at several tissue depths within the MdP, represented as isomorphic nested shells, shown in real positioning from an anterior-sagittal view. **(F)** Same nested surfaces as in **(E)** shown individually from a coronal view and without size differences. All panels except E shown from an anterior view (left = medial, right = lateral).

During development, tissue remodeling and cell behaviours reciprocally shape each other (Nestor-Bergmann et al., 2014). Directional cell behaviours can cause directional shape changes in tissues, while directional tissue growth can also induce organizing forces that orient cell behaviours along the growth axis (Heisenberg and Bellaïche, 2013; LeGoff and Lecuit, 2015). To study the balance of this relationship in the mandibular prominence, we compared spatial patterns of mitotic alignment at E10.5 to tissue growth in the half day before and after E10.5. We quantified mandibular shape at each age point (E10.0, E10.5, and E11.0) using seven manually placed landmarks, generated per-age shape atlases, and measured the shape change between each age (Figures 4A,B) (Lo Vercio et al., 2025). From E10.0-E10.5, outgrowth was concentrated in the antero-medial mandibular prominence (Figure 4C). This direction of outgrowth slowed in the half-day between E10.5 and E11.0, though outgrowth of the medio-rostral mandibular prominence slowed comparatively less than other parts of the tissue (Figure 4C). We visualized the average mitotic alignment score of neighbouring cells continuously across the E10.5 average shape and found that regions of well-aligned mitotic cells overlapped with areas of preceding (E10.0-E10.5) outgrowth (Figure 4D). Cells scored highest for mitotic alignment in the antero-medial domain of the mandibular prominence. We repeated this analysis in several nested shells representing different depths of the tissue. We found that mitotic alignment was medially biased at all depths, although the strength of the effect decreases deeper in the tissue, until the deepest layer becomes more polarized once again. (Figures 4E,F). Near the surface, the direction of cell divisions aligned closely with the primary direction of preceding tissue growth (Figures 3A, 4C,D).

### 2.5 Proliferation and positional mitotic orientation also follow mediolateral patterning

**FIGURE 5:**
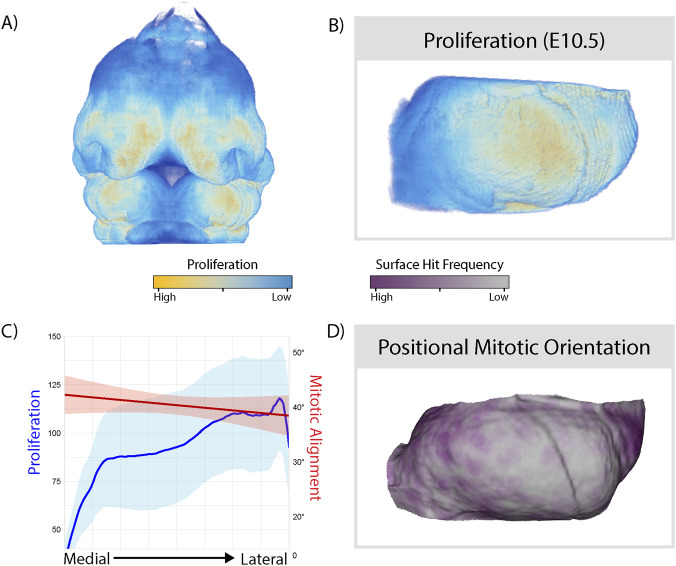
Mediolateral patterning of proliferation and cell-tissue interactions reveal shared coordination of cell behaviours in the MdP. Relative proliferation rates of all cells in the whole embryonic head **(A)** and left MdP **(B)** at E10.5. **(C)** Mean proliferation and mitotic alignment scores across the mediolateral axis of the left MdP at E10.5. Shaded areas represent standard error. **(D)** Frequency of ray-casted hits for positional mitotic orientation on the surface of the E10.5 left MdP. Purple regions indicate points on the tissue towards which more dividing cells orient their divisions. All panels shown from an anterior view (left = medial, right = lateral). Proliferation and positional mitotic orientation calculated for n = 4,520 cells from N = 4 specimens.

In addition to differences in mitotic alignment, we assessed whether spatial patterns in cell proliferation or cell-tissue relationships were associated with changes in shape. We generated whole-head and mandible-specific atlases of proliferation at E10.5 from an existing dataset (Lo Vercio et al., 2025) and registered them to our shape atlases (Figures 5A,B). We observed an inverse relationship between proliferation and mitotic alignment, in which cells in the medial tissue proliferated at a low rate but had more aligned division orientations, while cells in the lateral tissue divided more frequently and had less aligned orientations (Figure 5C). In addition, we calculated a positional orientation for each cell division, which describes the point on the tissue’s surface where the dividing cell points toward, rather than the absolute angle of division (Hanne et al., 2025). This accounts for the tendency of directional cell behaviours to orient themselves towards sources of mechanical cues, and reveals patterns present in the tissue that may be obscured by simply quantifying mitotic angle. Most cells oriented their divisions towards either the medial or lateral edges of the mandibular prominence, and the distribution of positional orientation across the surface was patchy (5D).

## 3 Discussion

**FIGURE 6:**
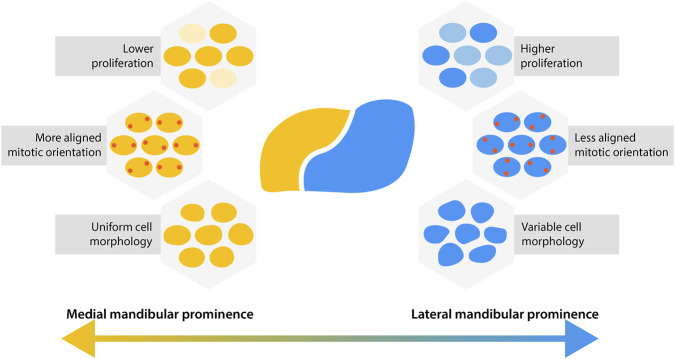
Two distinct domains of cell behaviours exist in the E10.5 mandibular prominence. Multiple cell behaviours follow shared mediolateral patterning in the MdP. The medial MdP contains cells that divide less frequently but align their divisions with their neighbours and exhibit more uniform cell morphology. The lateral MdP contains cells that divide at higher rates in random directions and exhibit more variable cell morphology.

In this study, we demonstrated cell behaviours in the mandibular prominence are patterned in shared mediolateral domains at E10.5, even though the tissue is structurally homogeneous (Figure 6). Cells in the medial mandibular prominence divide sparsely, but align their divisions well and are more morphologically similar. The lateral domain, conversely, generates bulk through densely dividing and poorly-aligned cells, which are more morphologically variable. Our findings suggest a relationship between the tandem coordination of multiple directional cell behaviours and oriented tissue outgrowth, specifically highlighting differences in mitotic orientation related to shape change.

We measured mitotic alignment in the developing jaw, which describes how similar the division angle of a cell is to those of its neighbours. Within the MdP, relatively large differences in mitotic alignment were observed across the mediolateral axis. Cells had higher mitotic alignment in the medial side of the tissue, especially at the superior edge of the MdP. Despite a lower proliferation rate in this region, tissue outgrowth was also greater in the medial side of the tissue. This suggests that mitotic alignment does not rely on high rates of proliferation to contribute to morphological patterning in the MdP. In subsequent days, the medial MdP will experience rapid outgrowth and narrowing towards the midline, which oriented divisions may help initiate (Higashiyama et al., 2025).

The cellular patterns described here demonstrate the important interplay between tissues and constituent cells. Mitotic alignment, proliferation rate, and cell morphology all followed similar mediolateral patterning at E10.5, suggesting a shared cue to establish domains of cell behaviour. Interestingly, these patterns aligned closely with patterns of outgrowth in the half day from E10.0-E10.5, but not from E10.5-E11.0. This suggests that directed tissue growth may help to maintain differences in cell behaviours by exerting coordinating forces on cells within, resulting in multiple cell behaviours under a common control.

The reciprocal roles of tissue growth and differential cell behaviours in establishing growth domains are consistent with previously established models of early mandibular development (Murillo-Rincón et al., 2025; Tao et al., 2019). Spatial differences in cell divisions are not sufficient to explain the contrasting narrow and bulbous domains of the mandibular arch; control of both tissue-scale properties and specifically oriented cell intercalations are required to establish distinct domains of growth (Tao et al., 2019). Differences in directional cell behaviours, such as rearrangements or oriented mitoses, create morphological contrast between parts of a tissue in both processes. Subsequent differences in tissue growth may then coordinate several cell behaviours to maintain distinct growth domains. This is exemplified by cell-tissue interactions, such as positional mitotic orientation. Most cells orient their divisions to either the medial or lateral extremes of the tissue, suggesting the presence of potential cues that maintain patterns of cell behaviours (Figure 5D). The patchy patterning of positional orientation also suggests the possibility of autocorrelation among cells, in which spatially proximate cells respond to cues together.

Positional information can come from either biomechanical or molecular sources. (Chan et al., 2017; Maroudas-Sacks and Keren, 2021; Urdy, 2012). However, regulation of the cellular basis of morphogenesis is better characterized in epithelial tissues than in mesenchyme (Fletcher et al., 2017; Panousopoulou and Green, 2014; Tao et al., 2019). Several key signaling pathways that influence directional cell behaviours, including canonical and non-canonical WNT/PCP signaling, are shared between both tissue types. In facial mesenchyme, WNT/PCP signals have roles in coordinating proliferation, migration, and cell polarity, which together influence growth patterns in morphogenesis (Hammond and Dixon, 2022; Marchini et al., 2021; Selleri and Rijli, 2023; Tang et al., 2016). These signals are most strongly expressed from the frontonasal prominence along with FGFs, but other signaling centers in the leading maxillary prominence (FGFs, BMPs) and the forebrain (SHH, BMPs, miRNAs) also influence facial outgrowth (Hu et al., 2003; Marchini et al., 2021; Marcucio et al., 2005; Trakanant et al., 2021; Xu et al., 2019). PDGFRα promotes early cranial neural crest migration into the facial prominences, where PDGFRβ contributes to cell proliferation (Dennison et al., 2021; Mo et al., 2020). However, it remains unclear how external cues orient cell division in mesenchymal tissues (Werts and Goldstein, 2011), despite mitotic orientation being the most strongly patterned directional cell behaviour identified in our study. Investigating whether established mechanical and molecular cues are associated with the mediolateral patterning of cell behaviours observed in this work will serve as important next steps.

Notably, the important role of tissue biomechanics in regulating cell behaviours is not fully addressed in this study, and remains a key focus for future research. Mechanical regulation from tissue properties directs cell decisions that ultimately influence the shape and proportion of the mandible (Du et al., 2021; Shwartz et al., 2012). Like most developing tissues, the early mandibular prominence is soft and fluid, but it stiffens as cells increase cortical actin crosslinking, helping to maintain the maturing tissue architecture (Du et al., 2021). Hippo/YAP signaling responds to mechanical changes to bridge mechanical and biochemical regulation of mandibular development, especially during later stages (Chen et al., 2021). Both mechanical and biochemical regulatory systems are active throughout development, and may contribute to the observed correlation between tissue growth and cellular coordination in this study.

These findings complement recent evidence that positional programs, rather than differences in cell identity, chiefly drive morphogenesis at early stages of development in mice. Several days before most cells in the face commit to their fates, positional cues drive differences in gene expression, even within generally homogeneous cell populations (Murillo-Rincón et al., 2025). Murillo-Rincón proposes that in early (E9.5-E11.5) facial mesenchyme, positional information is the primary driver of molecular heterogeneity. Our evidence aligns with this model and suggests that positional cues may be shared between cellular and molecular systems of regulation. The authors demonstrate spatial differences in gene expression in the E10.5 MdP that align closely with the cell behaviour patterns we observed. Specifically, the medial MdP expresses *Gsc*, while the lateral MdP shows expression of *Nr2f2* (Murillo-Rincón et al., 2025). Early positional information in soft mesenchyme may help establish the blueprint of later facial development, in which specific cell fates become the primary drivers of heterogeneity (Murillo-Rincón et al., 2025).

The head is a morphologically complex structure, driven by similar genetic and cellular complexity. Craniofacial morphogenesis requires intricate coordination of cell behaviours at all spatial and temporal scales of development. Perturbation at any step in the development of facial tissues can persist throughout the entire process, resulting in structural birth defects such as micrognathia. Many facial phenotypes, including micrognathia, can arise from numerous genetic or environmental causes, and often have poorly defined or unknown origins (Bromley and Benacerraf, 1994; Paladini, 2010; Price et al., 2016). However, similar phenotypes may be driven by similar alterations in the patterning of cell behaviours, even when the molecular or environmental causes are distinct. This makes facial structures such as the mandibular prominence excellent models for understanding how cell behaviours structure the growth of complex tissues. The fundamental principles that govern patterning of cell behaviours may have broad implications across normal development and diverse health contexts. A robust understanding of these principles requires further investigation not only into the morphogenic potential of cell behaviours, but also the positional information by which molecular and cellular patterns are established.

## 4 Materials and methods

### 4.1 Animal generation

This study used embryos generated from wild-type C57Bl6/J mice from The Jackson Laboratory and bred at the University of Calgary. All animal work was approved by the University of Calgary IACUC (B.H.). Mice were maintained on a 12-h light/dark cycle, and embryos were collected 10.5 days following observation of a post-coital plug. Dams were anesthetized by 5% vaporized inhalant isoflurane to the deep/surgical plane of anesthesia, as determined by absent toe and eye reflexes, in accordance with our AUP, then euthanized by cervical dislocation for embryo collection.

### 4.2 Clearing and staining

Following euthanasia and collection, embryos were washed for 30 min in PBS to remove blood, then fixed overnight in 4% paraformaldehyde in PBS at 4 °C. Embryos were cleared with a modified CUBIC protocol based on (Susaki et al., 2015) to preserve morphology while removing light-obstructing heme, pigments and lipids. Atypically developing embryos were excluded from analysis.

During the clearing protocol, samples were washed in PBS for 24 h after fixation, then serially dehydrated in increasing concentrations of methanol in PBS (25%, 50%, 75%, 100%) for a minimum of 30 min each. If needed, samples were stored long-term in 100% methanol. Samples were then permeabilized in 5% H_2_O_2_ in methanol overnight at 4 °C. Samples were then rehydrated in decreasing concentrations of methanol in PBS (100%, 75%, 50%, 25%, 0%) for a minimum of 30 min each, with an additional 30m PBS wash. Embryos were cleared in CUBIC-1 (25% urea, 25% Quadrol, 15% Triton X-100). To transition into clearing, samples were first incubated overnight in 1:1 PBS:CUBIC-1, then fully cleared in 100% CUBIC-1 at 37 °C while shaking until completely transparent (∼5–10 days). Clearing was then halted by rinsing twice in PBS for 2h, then once more overnight. Samples were then blocked for 36 h in 5% goat serum, 5% DMSO in 0.1% sodium azide in PBS at 37 °C to prevent nonspecific binding (Lo Vercio et al., 2025; Susaki et al., 2015).

For immunostaining, samples were incubated in anti-phospho-Aurora A rabbit primary antibody (Invitrogen, 44-1210G; 1:200) for 5 days, then Alexa Fluor® 555 donkey anti-rabbit secondary antibody (Invitrogen, A31572; 1:5,000) for spindle pole staining together with CS Nuclear Green (1:4,000) for Nuclear staining for an additional 5 days. Antibodies were diluted in 5% DMSO in 0.1% sodium azide in PBS and incubated 37 °C while shaking. Following both primary and secondary staining, samples were washed several times in 0.5% Tween-20 in PBS to remove excess antibody. Heads and bodies of stained samples were separated prior to imaging; both were then embedded in 1.5% agarose, dehydrated in 30% sucrose/dH_2_O for 30 min, and incubated in CUBIC-2 (25% urea, 50% sucrose, 10% tri-ethanolamine in PBS) for 24 h.

Phospho-Aurora A is a useful marker of mitotic spindle orientation due to its temporal specificity; it is absent during most of the cell cycle and peaks expression during metaphase, at which time the cell is already aligned along the mitotic plane. Marked spindle poles are therefore highly likely to be informative of the cells mitotic orientation(Lukasiewicz et al., 2011; Van Horn et al., 2010).

### 4.3 Imaging

Light-sheet images of samples embedded in agarose columns were obtained with a Zeiss Lightsheet Z.1 microscope. Samples were submerged in CUBIC-2 in the imaging chamber and rotated to minimize light scattering in the left MdP prior to imaging. 488nm (nuclei) and 561 nm (phospho-Aurora A) were activated in a single track, allowing image capture in both channels simultaneously. Optical slices were taken every 1.07 µm with a 0.2 µm in-plane pixel resolution using a ×20 immersion objective immersed in CUBIC-2. Tails were imaged separately at lower magnification, with slices every 7 µm and a 2.5 µm resolution; embryos were staged by manually counting tail somites and assessing limb bud morphology (Theiler, 1989). Image stacks of the front-face, including the maxillary, mandibular, and frontonasal prominences, were captured with a 10% overlap and automatically re-stitched in the ZEN Black imaging software (Zeiss). All light-sheet images were then exported as TIFF stacks in ZEN Blue image processing software (Zeiss) for further analysis.

### 4.4 Image segmentation

Nuclear segmentation was based on a pipeline previously published from our group using convolutional neural networks to segment nuclei from LSFM images (Lo Vercio et al., 2022; 2025). However, the higher scanning resolution of the data in this publication required the training of a new model. Nuclei were therefore automatically segmented using CellPose-SAM and fine-tuned in-the-loop on 8 images (4096 × 4096) from E10.5 embryos depicting the Nuclear Green channel (Pachitariu et al., n.d.; Stringer et al., 2021). An expert observer iteratively corrected CellPose-SAM’s segmentation of the training images until satisfactory performance was achieved. Each image within the test set was annotated by two expert observers to compare performance to inter-observer agreement. The inter-observer DICE score for the test set was 0.83, and the DICE score between the automated segmentation and manual annotation was 0.86.

Spindle poles were manually identified and paired across the cell body. Due to the anisotropy of LSFM imaging, spindles could not be reliably paired between planes. Therefore, only spindle pairs captured entirely within the imaging plane were included. To ensure our results were still applicable to a 3D context, images taken in multiple orientations were included to ensure diverse directions of spindle orientation were captured. Two expert observers annotated spindle poles in every 5^th^ slice (5.4 µm interval) of the mandibular prominence in each image. These annotations were directly processed through CellProfiler as described below to extract cellular features. Additionally, spindle pole annotations were used to filter mitotic nuclei from the nuclear segmentation by pairing each spindle pole annotation with its nearest neighbour from the segmented nuclei.

### 4.5 Mitotic orientation and cell morphology

A mitotic angle was calculated for each spindle orientation vector in relation to the rostral side of the face. To do this, we applied a pipeline in CellProfiler (Version 4.2.1) to each annotated spindle pair to extract mitotic orientation and associated cellular features (Stirling et al., 2021). Mesenchymal cell divisions are symmetrically bidirectional (i.e., there is no ‘front’ or ‘back’, and vectors pointing in either direction are effectively the same angle). As such, each mitotic angle was represented by its ‘head’ ranging from 0° to 180°, and angle doubling was used to calculate means and distributions of angles. For analysis of mitotic orientation across biological axes, tissues were divided into six segments along the mediolateral axis to allow for better spatial resolution. In addition to absolute mitotic orientation, we calculated a contextual mitotic alignment score for each cell, which represents the angular difference between a cell’s mitotic angle and the average mitotic angle of all cells in a 200 µm 3D radius around it. Mitotic alignment scores range from 0° to 90°, with lower scores indicating that a cell is dividing in a similar direction to those around it. A total of 4,520 mitotic cells were analysed from 4 samples.

Angle distributions were analyzed with the Circular package in R (Lund et al., 2025). We used the circular Kuiper’s test to assess whether angle distributions deviated from randomness, and Watson’s U^2^ test to compare distributions between cell groups, as well as between mitotic and nuclear orientations.

To study morphological differences between cells in directionally growing tissues, we used CellProfiler to extract 5 geometric parameters from the nuclear shapes of each dividing cell: area, perimeter, aspect ratio, eccentricity, and long axis orientation. Nuclear shape is more resistant to light-scattering perturbations in large, 3D tissues with LSFM compared to cell surface shape, and closely approximates the shape of the constituent cell in actively mitotic tissues (Chen et al., 2015; Lawton et al., 2024; 2024; Li et al., 2015). To assess high-level variation in geometric features, we conducted principal components analysis (PCA) on the normalized values of these parameters.

### 4.6 Shape analyses

Because the shape of the mandibular prominence changes dramatically across embryonic time points, separate shape atlases were created for each age studied (E10.0, E10.5, and E11.0) using MusMorph webtools, available at hallgrimssonlab.ca (Devine et al., 2022). Following rigid alignment and registration with ALPACA+, each atlas was generated as the average of 5 samples, with each contributing a left prominence and its mirrored right counterpart (Porto et al., 2021).

3DSlicer was used to generate 3D surface meshes from segmented mesenchymal volumes for all samples. We identified 7 landmarks that could be placed at all embryonic age points studied (Supplementary Table S2; Figure 4B) and manually applied these landmarks to all sample meshes. The same landmark scheme was also used to manually place landmarks on the average meshes (i.e., atlases) for each time point.

We used ordinary Procrustes analysis to superimpose all landmark coordinates onto the E10.5 atlas and scale the configurations to a unit centroid size (Dryden, 2025). To visualize shape differences across developmental stages, we morphed the atlas meshes to their respective mean landmark configurations. Heatmaps of shape differences were then generated using meshDist, plotting the closest-point distances between two average surface meshes (e.g., E10.0-E10.5) (Schlager et al., 2025).

For an additional subset of our sample mandibles that also had cell behaviour data (n = 4), we extracted the transformation matrix from the Procrustes analysis. We then applied this matrix to the coordinates and angles of the corresponding spindle orientation vectors, converting all angles to a common coordinate space. Mitotic angles were converted to unit vectors and decomposed into their x, y, and z components before transformation to allow for 3D analysis.

To relate patterns of mitotic orientation and alignment with shape change, the atlas meshes were decimated, and at each vertex, we calculated and plotted the average mitotic alignment and mitotic orientation vector of all cells within a 200 µm radius. However, these plots only represent the outer surface of the mandibular prominence, and cells deeper within the tissue may also contribute to the observed growth patterns. To account for this, we generated nested isomorphic shells at multiple tissue depths and calculated cell behaviours using the same approach described previously (Figure 4E).

## Data Availability

The processed datasets generated for this study are publicly available and can be found in the University of Calgary Data Repository (https://doi.org/10.5683/SP3/SNGOJC). Due to large file size, the original image data are available upon request. Additional existing data used in this study can also be found in the University of Calgary Data repository (https://doi.org/10.5683/SP3/AXMCDE). The source code produced and used in this work can be found in GitHub (https://github.com/Hallgrimsson-lab/mandibular-morphogenesis-2026).
