# Supplementary material for: Three-dimensional cellular dynamics and mandibular morphogenesis
Source: Front Cell Dev Biol. 2026 May 18;14:1823297. doi: 10.3389/fcell.2026.1823297 (PMC13223115; doi:10.3389/fcell.2026.1823297)
Supplement: Supplementary file 1 [file DataSheet1.docx]

**Supplementary Information**

Supplementary Table 1. Correlation between individual measures of nuclear morphology, mitotic alignment, and mediolateral position.

| Feature of Nuclear Morphology | Alignment | | Mediolateral position | |
| --- | --- | --- | --- | --- |
|  | Correlation | p-value | Correlation | p-value |
| Cross-sectional area | -0.035 | 0.29 | -0.017 | 0.62 |
| Eccentricity | -0.038 | 0.25 | -0.131 | <0.001* |
| Mean Radius | -0.026 | 0.44 | 0.045 | 0.18 |
| Perimeter | -0.049 | 0.15 | -0.023 | 0.49 |
| Aspect Ratio | -0.039 | 0.25 | -0.132 | <0.001* |

Supplementary Table 2. Landmark positions for the E10.0-E11.0 murine mandibular prominence

| Landmark ID | Anatomical Description |
| --- | --- |
| 1 | Anteriormost intersection of the left and right mandible in the midline |
| 2 | Posteriormost intersection of the left and right mandible and second pharyngeal arch in the midline |
| 3 | Maximum point of curvature where the mandible contacts or comes closest to the maxilla |
| 4 | Rostral maximum of mandibular prominence |
| 5 | Lateralmost maximum of the mandibular prominence |
| 6 | Posteriormost intersection of the mandibular prominence and second pharyngeal arch on the lateral side |
| 7 | Posteriormost corner of the mandibular mass and mandibular waist, on the lateral edge |

**
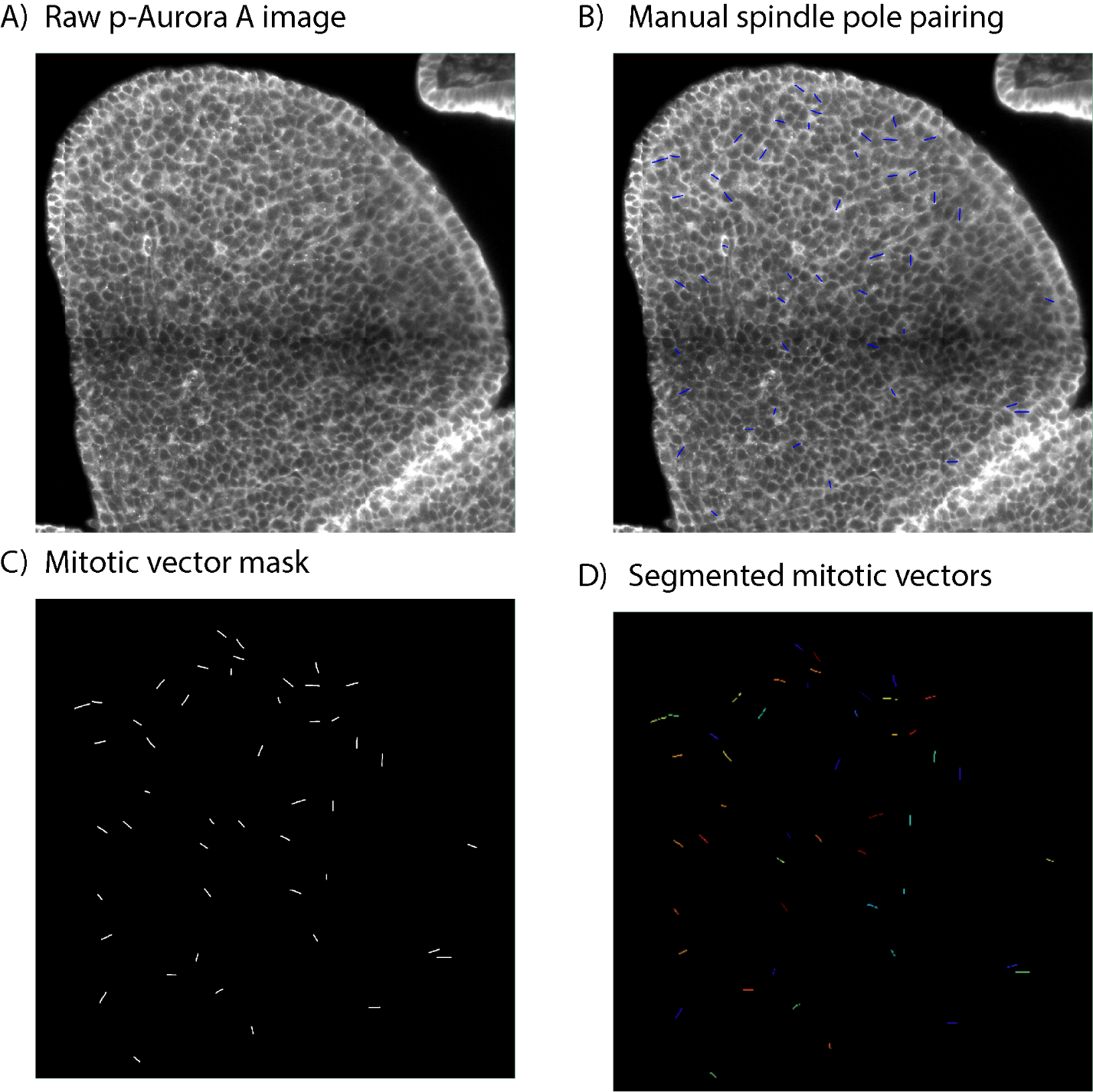
**

Supplementary Figure 1. Quantification of mitotic vectors from phospho-Aurora A LSFM data. A) Spindle poles are marked by small, bright punctae within cell bodies. B) Spindle poles are manually paired across cell bodies by an expert observer. These annotations are also used to filter mitotic nuclear geometry. C) Annotations are converted into a binary mask for analysis in CellProfiler. D) Input masks are downsampled and each object is identified as an individual mitotic vector. Long-axis orientation is used as a measure of mitotic angle in 2D within the imaging plane, then transformed into a common three-dimensional space.
